# A call for increased measurement of eating disorders and disordered eating in federal surveillance in Canada

**DOI:** 10.24095/hpcdp.45.6.04

**Published:** 2025-06

**Authors:** Amanda Raffoul, Maria Nicula, Chloe Gao, Nicole Obeid

**Affiliations:** 1 Department of Nutritional Sciences, Faculty of Medicine, University of Toronto, Toronto, Ontario, Canada; 2 Department of Health Research Methods, Evidence, and Impact, Faculty of Health Sciences, McMaster University, Hamilton, Ontario, Canada; 3 Department of Medicine, The University of British Columbia, Vancouver, British Columbia, Canada; 4 Eating Disorder Research Lab, Children’s Hospital of Eastern Ontario Research Institute, Ottawa, Ontario, Canada

**Keywords:** feeding and eating disorders, body image, public health surveillance, health policy, nutrition policy

## Abstract

Eating disorders (EDs) and disordered eating present a significant health burden given their prevalence and associated health risks; however, there are notable gaps in population-level surveillance of EDs and disordered eating in Canada. These data gaps limit our understanding of the scope of the problem and present challenges to monitoring trends in EDs and disordered eating in response to changing health and policy contexts, such as the COVID-19 pandemic. We screened Canadian federal health surveillance surveys to identify measures of ED diagnosis, engagement in disordered eating behaviours (e.g. binge eating, self-induced vomiting) and related constructs (e.g. weight perception, body satisfaction). Among adults, there was a 10-year gap in ED measurement, and there has been no assessment of engagement in any type of disordered eating behaviours. Among children and adolescents, there have been recent improvements in the measurement of disordered eating behaviours, but there are no surveys that include measures of binge eating, the most common disordered eating behaviour. National surveillance data assessing EDs and disordered eating are necessary to quantify their burden, assess trends in relation to evolving health and policy contexts and identify individuals who face barriers to seeking treatment services. We conclude by providing recommendations for constructs that should be measured, as well as guidelines for measurement development in conjunction with community members and clinical and research experts.

**Highlights**

- Although the public health burden of eating disorders is significant, little is known of their prevalence in Canada.
- Canadian federal surveillance health surveys do not accurately assess eating disorders and disordered eating across age groups.
- The exclusion of eating disorders and disordered eating from federal health surveillance has important, negative consequences on Canadian public health and health policy, including the inability to track incidence in response to exacerbating events or to assess differences among groups that are often marginalized.
- Eating disorders must be adequately represented, monitored and tracked in Canadian federal surveillance using items that are informed by research, clinical and community partners.

## Introduction

Eating disorders (EDs) are severe psychiatric illnesses that present an often neglected and growing public health burden.^1^ EDs, including anorexia nervosa (AN), bulimia nervosa (BN), binge eating disorder (BED), other specified feeding or eating disorder (OSFED) and avoidant/restrictive food intake disorder, carry an estimated international point prevalence between 2.2% and 4.6%.^2^ Although the case mortality rate for AN is among the highest of any psychiatric illness, second only to that of opioid use,^3^ it is important to note that all EDs present significant risks to the mental, physical and emotional well-being of those affected^4^ as well as those caring for them.^5^

“Disordered eating” encompasses attitudes and behaviours that may not meet the diagnostic threshold for an ED but still present risks to population health, such as excessive exercise; extreme caloric restriction; self-induced vomiting; the misuse of diuretics, laxatives and muscle-building and weight-loss products; and binge eating. A key risk factor for the development of EDs, disordered eating is also associated with poor cardiometabolic outcomes and worsened mental health.^6^,^7^

Globally, there were an estimated 55.5 million cases of EDs in 2019, with BED and OSFED accounting for the most cases.^8^ In Canada, the prevalence of EDs depends on the sample studied—one survey of 3043 adolescents found that 2.2% to 4.5% met diagnostic criteria for ED,^9^ another study of a community-based sample found that 8% to 15% of participants aged 15 to 71 years reported clinically significant ED disturbances,^10^ while data from the nationally representative 2002 Canadian Community Health Survey (CCHS) found that 0.47% of the population self-reported an ED diagnosis.^11^ One meta-analysis of studies from 16 countries revealed that 22% of children and adolescents engage in disordered eating,^12^ and survey studies of community-based (i.e. nonclinical) samples of children and adolescents in Canada have estimated similar prevalences.^10^,^13^-^15^

However, the prevalence of EDs and disordered eating in Canada are likely grossly underestimated for several reasons. First, there is a common misconception that EDs only affect a small minority of the population; however, a growing body of literature now highlights disparities in ED screening, identification and diagnosis by weight, gender, racialized identity, ethnicity and income.^16^,^17^ Therefore, the use of clinical data sources or the reliance on self-reported diagnosis likely leads to underestimation of the actual prevalence of EDs, particularly among marginalized populations.^18^ Further, in Canada, ED research is significantly underfunded relative to health care utilization costs and other mental health conditions,^19^ limiting researchers’ ability to conduct representative cross-sectional and cohort studies that can more accurately estimate prevalence, incidence and severity of behaviours. 

Finally, EDs and disordered eating do not exist in a vacuum, but rather are heavily influenced by broader health and policy contexts, including changes to health care administration, evolving food environments and external stressors, such as the COVID-19 pandemic. In fact, the COVID-19 pandemic led to significant increases in emergency department visits and hospitalizations for Canadians across the lifespan that have not yet returned to prepandemic levels.^20^ There are massive gaps in population-level surveillance of EDs and disordered eating, which limits our knowledge of the scope of the problem and precludes our ability to monitor trends over time, especially given shifts seen since the onset of the COVID-19 pandemic.^21^ Despite these limitations, there have been some federal-level efforts to capture ED presentations and disordered eating symptoms.

## Mapping the landscape of eating disorder surveillance in Canada

To assess the landscape of ED-related measures in Canadian federal surveillance, we screened health surveys conducted or supported by Statistics Canada and the Public Health Agency of Canada.^22^ We aimed to identify measures of self-reported ED diagnosis; self-reported engagement in disordered eating, including binge eating, self-induced vomiting and the use of diet pills; weight or appearance modification efforts, such as attempts to lose, gain or maintain weight; and related constructs, including weight perception and body or weight satisfaction. Five surveys were identified through this process: the CCHS, the Canadian Health Measures Survey (CHMS), the Canadian Health Survey on Children and Youth (CHSCY), the Mental Health and Access to Care Survey (MHACS) and the Health Behaviour in School-aged Children (HBSC). A summary is presented in Table 1.

**Table 1:** Overview of measures assessing disordered eating, eating disorders and related constructs in federal surveillance

Construct	Year(s) – survey name^a^	Measure details (response options)
Self-reported eating disorder diagnosis	2012 – CCHS Mental Health 2022 – MHACS	“Do you have an eating disorder such as anorexia or bulimia?” (Y/N)
Parent-reported eating disorder diagnosis	2016 – CHSCY 2019 – CHSCY 2023 – CHSCY	“Does [your child] have an eating disorder such as anorexia nervosa or bulimia?” (Y/N) Age of diagnosis was also assessed
Eating disturbances	2002 – CCHS Mental Health and Well-being	Participants who answered “yes” to both questions were presented with the full Eating Attitudes Test (EAT-26)^23^ (1) “Was there ever a time in your life when you had a strong fear or a great deal of concern about being too fat or overweight?” (Y/N) (2) “During the past 12 months, did you have a strong fear or a great deal of concern about being too fat or overweight?” (Y/N)
Weight perception	2012 – CCHS Mental Health 2010 and subsequent iterations – CCHS Annual Component 2016 – CHSCY (parent and child) 2007 and subsequent iterations – CHMS	“Do you consider yourself...?” (Overweight/Underweight/Just about right)
	2001/02 – HBSC 2009/10 – HBSC 2013/14 – HBSC 2017/18 – HBSC	“Do you think your body is…?” (Much too thin/A bit too thin/About right/A bit too fat/Much too fat)
Body/weight satisfaction	2010 and subsequent iterations – CCHS Annual Component	“How satisfied are you with the way your body looks?” (Very satisfied/Satisfied/Neither satisfied nor dissatisfied/Dissatisfied/Very dissatisfied)
	2016 – CHSCY	“How often are you satisfied with your weight?” (Never/Rarely/Sometimes/Often/Always)
General weight modification efforts	2016 – CHSCY 2019 – CHSCY 2023 – CHSCY	“In the past 12 months, how often have you … Changed your eating habits in order to manage your weight?” (Never/A few times/Monthly/Weekly/Daily)
	2001/02 – HBSC 2005/06 – HBSC 2009/10 – HBSC 2013/14 – HBSC	“At present are you on a diet or doing something else to lose weight?” (No, weight fine/No, need to lose/No, need to put on/Yes)
Preoccupation with thinness	2016 – CHSCY 2019 – CHSCY 2023 – CHSCY	“In the past 12 months, how often have you … Been preoccupied with a desire to be thinner?” (Never/A few times/Monthly/Weekly/Daily)
Self-induced vomiting	2016 – CHSCY 2019 – CHSCY 2023 – CHSCY	“In the past 12 months, how often have you … Vomited to lose weight?” (Never/A few times/Monthly/Weekly/Daily)

**Abbreviations**: CCHS, Canadian Community Health Survey; CHMS, Canadian Health Measures Survey; CHSCY, Canadian Health Survey on Children and Youth; HBSC, Health Behaviour in School-aged Children; MHACS, Mental Health and Access to Care Survey; Y/N, yes/no. 

^a^ The sample for CCHS consists of persons aged 18 years and older living in the 10 provinces and the three territories. The sample for MHACS consists of persons aged 15 years and older living in the 10 provinces. The sample for CHSCY consists of persons aged 1 to 22 years (and their guardians) living in the 10 provinces. The sample for CHMS consists of persons aged 1 to 79 years living in the 10 provinces. The HBSC study is a World Health Organization research study that focusses on the health and well-being of young people aged 11 to 15 years. 

Among adults, self-reported ED diagnosis was measured in the 2012 CCHS – Mental Health component and the 2022 MHACS, leaving a 10-year gap in ED observations. Parent-reported eating disorder diagnosis and age of diagnosis were included in the 2016, 2019 and 2023 iterations of the CHSCY. The items used to assess self-reported ED diagnosis in these surveys all query whether the affected person has “an eating disorder such as anorexia or bulimia,” despite BED and OSFED being the most common EDs.^8^ This limits the construct validity of the question for the assessment of EDs more broadly and the ability of researchers to know the true prevalence of EDs in Canada. Instead of ED diagnosis, the 2002 CCHS Mental Health and Well-being survey used a 2-item screener to assess whether respondents should fill out the 26-item Eating Attitudes Test,^23^ a comprehensive assessment of eating disturbances that has not been administered since.

Disordered eating is severely undermeasured, as there has been no federal surveillance of engagement in any type of disordered eating behaviours among Canadian adults. The development of the CHSCY, first piloted in 2016 and implemented in 2019 and 2023, is a positive advancement for tracking disordered eating among youth; however, even this survey only incorporates the assessment of one behaviour—self-induced vomiting—alongside an attitudinal measure of preoccupation with thinness. There are no surveys, either of children or adults, that include measures of binge eating, the most common disordered eating behaviour.^24^ Further, there are no known measures of the misuse of dieting-related products, including laxatives and natural health products (NHPs) with claims related to weight-loss and muscle-building, which have been associated with an increased prospective risk of disordered eating and EDs among adolescents and young adults.^25^,^26^

Additional ED-related constructs, including weight perception, weight satisfaction and general weight modification efforts have been assessed through single-item measures in several surveys (Table 1). Notably, the items focus on weight satisfaction rather than satisfaction with overall appearance, body shape and muscularity, which may underestimate body dissatisfaction among boys and men,^27^ or even the general population as appearance ideals shift over time.^28^ Relatedly, the disparate measurement of constructs across surveys limits our ability to assess longitudinal trends in outcomes related to EDs and disordered eating. Although the HBSC, CCHS and CHSCY include recurring measures over at least two waves, no one survey alone provides a true picture of the population prevalence or incidence of EDs and disordered eating in Canada.

## Public health and policy implications

The gaps in national surveillance of EDs and disordered eating result in several consequences. First, national data are needed to quantify and make visible the burden of EDs. As the common health policy truism states, “if something cannot be measured, it cannot be improved.”^29^ Accordingly, a better understanding of the prevalence of EDs is needed to dispel pervasive myths about their low prevalence and the affected populations,^30^ to better estimate related costs^21^ and to support clinical and research funding that matches the burden they represent.^20^

Relatedly, if EDs and disordered eating are not measured across representative national samples, then we will fail to assess trends in relation to rapidly changing health and policy contexts. Changes to health care policy and administration may lead to changes in the incidence of EDs that cannot be assessed without adequate surveillance. For example, the introduction of Ontario’s Eating Disorders quality standards^31^ to guide services will require the ability to measure how their implementation may shift ED incidence in the province. 

As well, external population-level stressors, such as the COVID-19 pandemic, may exacerbate the burden of EDs and disordered eating on health care systems and society more broadly. Indeed, a recent analysis of the impact of the pandemic on ED-related costs in Canada specifically highlighted that the lack of adequate surveillance data limited their analyses and makes any future attempts at costing studies futile until better measurement of prevalence is in place.^21^

Ongoing food policy changes, including Canada’s Healthy Eating Strategy,^38^ may have the potential to heighten or ameliorate disordered eating among populations. For example, concerns have been raised about the potential of mandatory calorie labelling on menus to worsen disordered eating pathology among vulnerable people,^32^ but the impact of this policy on population-level disordered eating incidence over time is challenging to appraise. In light of newly introduced food environment legislation, it is necessary to assess the impacts of nutrition policy on disordered eating in the population.

Finally, improved federal surveillance of disordered eating and EDs is a matter of health equity. Relying on diagnosis alone through health administration databases (e.g. those of the Canadian Institute for Health Information, the Institute for Clinical Evaluative Sciences) limits our knowledge of who is affected because of known barriers to diagnosis and accessing treatment. It also does not take into account the estimated 76.8% of those with a diagnosable ED who never receive care.^33^ Therefore, a dependence on clinical administrative data sources leads to the insufficient and inconsistent inclusion of those who are less likely to seek out and obtain care, including individuals with higher weights, boys and men, older adults and racialized people.^17^,^18^

Health equity is important to consider relative to disordered eating as well. Several disordered eating behaviours, including binge eating and the use of unprescribed weight-loss NHPs, are significantly more prevalent among populations who experience elevated discrimination.^34^,^35^ The exclusion of binge eating from surveillance surveys is especially noteworthy, as it is more common among racialized populations and individuals with higher weights relative to their peers,^36^,^37^ limiting our ability to assess inequities in disordered eating engagement.

## Conclusions and recommendations

EDs and disordered eating are not adequately measured by Canadian federal surveillance, which presents significant implications for public health and policy evaluation in Canada. The lack of surveillance data also limits the research of population health scientists who are interested in these topics, which subsequently leads to less funding and a lower priority in Canada’s health funding sphere. Comprehensive and representative assessments of EDs and disordered eating are aligned with Canada’s current strategies to improve healthy eating and food environments,^38^ as well as the recently announced CAD 500 million Youth Mental Health Fund.^39^ EDs, which historically have been left out of federal mental health discourse, require adequate monitoring to ensure that this renewed commitment to youth mental health does not neglect their ongoing burden.

There are several different parameters relative to EDs and disordered eating that can be assessed. We recommend the inclusion of all of the following items on assessment instruments, where possible:

- **self-reported ED diagnosis,** as a single item but listing several diagnoses, including BED and OSFED, in addition to AN and BN;
- **binge eating,** which should always be included in the assessment of disordered eating behaviours, given its prevalence, high burden among diverse populations and associations with a range of physical and emotional well-being outcomes;
- **restrictive disordered eating behaviours,** including fasting or skipping meals, as well as engagement in diet programs that eliminate or restrict food groups (e.g. a no-carb diet);
- **compensatory disordered eating behaviours,** including weight-loss and muscle-building NHP use, laxative misuse and excessive exercise; and
- **psychological contributors to disordered eating,** including body dissatisfaction, weight-based bullying and preoccupation with body weight or shape.

The wording of measures, including their assessment of frequency (e.g. binge eating in the past 30 days vs. the past 3 months), should be decided and co-developed with community members, clinical experts and research experts depending on the survey. For example, the US-based Youth Risk Behavior Survey (YRBS) enquires about past–30 day engagement in disordered eating behaviours to align with survey measures for other health behaviours (e.g. smoking, vaping).^40^

We also recognize the challenges that decision makers encounter in developing federal surveillance surveys, including the costs associated with measurement development and testing, survey respondent burnout, and shifting governmental health priorities. The adequate measurement of EDs and disordered eating using brief or even single items is challenging but can be done through expert- and community-informed measure development, the latter of which can bolster survey response rates and increase the uptake of study results.^41^

For example, after the US Centers for Disease Control and Prevention removed disordered eating items from the nationally representative YRBS in 2015, a grassroots collective of ED experts launched a working group that generated evidence-based recommendations for the inclusion of brief disordered eating measures.^40^ This list of measures is tailored to YRBS developers and includes specific recommendations for the inclusion of one to four questions, depending on availability. The group recommends the use of composite measures (e.g. combining multiple behaviours into a single item), which can be used to minimize survey length and participant burden. As a result of their efforts, the national 2024 YRBS now includes a single item assessing binge eating in its nationally representative survey.^40^ State representatives can also choose to elect to add one or more of the following: a composite measure of disordered eating behaviours (i.e. fasting or skipping meals, taking diet pills or supplements not prescribed by a doctor, or vomiting or taking laxatives); a measure assessing weight victimization; and/or a series of three independent questions enquiring about the frequency of disordered eating behaviours. 

ED researchers and advocates in other countries, such as Australia,^30^ have made similar calls to improve surveillance efforts. To allow for cross-country comparison, where possible, we encourage global collaboration in measure development and the creation of common minimum measures.

EDs and disordered eating present significant challenges to health care systems and population health in Canada. Adequate and evidence-informed monitoring of EDs and disordered eating are sorely lacking in federal health surveillance but are needed to address the urgent and growing burden of these conditions.

## Acknowledgements

This research received no specific grant from any funding agency. AR is supported by a William T. Grant Foundation Scholars Award (SCH-204232). CG is supported by a Canadian Institutes of Health Research Vanier Graduate Scholarship and a University of British Columbia Friedman Award for Scholars in Health.

## Conflicts of interest

The authors have no conflicts of interest to disclose.

## Authors’ contributions and statement

AR, MN: conceptualization.

AR, MN, CG: investigation, visualization.

AR: writing—original draft, supervision.

AR, MN, CG, NO: writing—review and editing.

The content and views expressed in this article are those of the authors and do not necessarily reflect those of the Government of Canada.
